# A Qualitative Analysis of Trialogues Between People with Lived Experience, Their Relatives, and Mental Health Professionals

**DOI:** 10.1007/s10597-024-01402-3

**Published:** 2024-12-06

**Authors:** Francisco José Eiroa-Orosa, María Incera-Rosas

**Affiliations:** 1 Section of Personality, Evaluation, and Psychological Treatment, Department of Clinical Psychology and Psychobiology, School of Psychology, Institute of Neurosciences, University of Barcelona, Barcelona, Catalonia Spain; 2 First Person Mental Health Research Group, Veus, Catalan Federation of First Person Mental Health Organisations, Barcelona, Spain

**Keywords:** Community-based participatory research, Recovery, Mental health

## Abstract

Trialogue meetings, aligned with the Recovery model in mental healthcare, foster the development of a shared language and mutual understanding among persons with lived experience, their relatives, and professionals. This study analysed the first mental health trialogue experience in Barcelona, involving six meetings with thirteen voluntary participants over three months. Using qualitative methods for data collection and reflexive thematic analysis, three main themes emerged: Recovery process, Interaction with mental health services, and Trialogue dynamics. Benefits included open dialogues in a safe space, learning from diverse perspectives, and sharing expertise. The meetings created an environment of empathy and respect while promoting open communication. This approach holds substantial potential for transforming mental healthcare systems, suggesting trialogues can be an effective tool for enhancing communication within community-based mental health initiatives. The findings provide a comprehensive picture of the trialogue process, highlighting its potential to foster mutual understanding among participants.

**Supplementary Information:**

The online version contains supplementary material available at 10.1007/s10597-024-01402-3.

## Introduction

Over recent years, the need for a more inclusive mental health system has grown, with the Recovery model (Anthony, 1993) becoming increasingly relevant (Davidson, 2016; Slade et al., 2014). This model, which emerged from various social movements involving individuals with lived experience of severe psychosocial distress, their relatives, and mental health professionals, opposes biomedically oriented approaches focused on symptom remission (Woods et al., 2022). Instead, it focuses on living a satisfying and hopeful life despite experiencing symptoms of mental distress, emphasising person-centred care, self-esteem, empowerment, and personal growth. Recovery involves changing attitudes, values, and skills, and promotes participatory decision-making (Davidson et al., 2020). This approach acknowledges the expertise of individuals with lived experiences of severe psychosocial distress, encourages community inclusion, challenges stigma and discrimination, and supports self-management (Slade et al., 2014). Recent reviews show how various Recovery-oriented interventions and programs, such as training and awareness for mental health professionals (Eiroa-Orosa & García-Mieres, 2019), peer support (Cooper et al., 2024), wellness Recovery action planning (Canacott et al., 2019), or mental health trialogues (Mac Gabhann & Dunne, 2021), have developed into effective interventions that foster horizontal discursive practices and can break down institutional barriers.

A trialogue is a community-building activity involving persons with lived experience, relatives, and mental health professionals, aimed at establishing open and inclusive dialogues among stakeholders with equal voices in the conversation (Amering et al., 2002). While the primary purpose is to foster a sense of community and mutual understanding, trialogue meetings can also lead to transcending role stereotypes. Through these exchanges, all three groups gain insights, knowledge, and practical skills for collaborative daily interactions. Participants convene to engage in open discussions on equal footing (Amering et al., 2012). Each participant brings expertise through personal experience, professional training, or both.

Trialogue meetings are deeply rooted in the principles of Open Dialogue, a therapeutic approach developed in Western Lapland, Finland, in the 1980s. Open Dialogue, influenced by Bakhtin’s (1981) dialogic theory, emphasises creating a space where all voices are equally valued. This approach supports meaningful conversations that can lead to personal and relational transformation. The origins of trialogic practices can be traced back to the Psychosis Seminars initiated in Hamburg, Germany in the late 1980s (Bock & Priebe, 2005). The term ‘seminar’ was deliberately chosen to underscore the mutual learning among participants. Dorothea Buck, who was interned and psychiatrised in Nazi Germany just before the Second World War and thereafter, shared her insights into the dehumanising aspects of psychiatric care. She notably emphasised that individuals should never be deprived of their worth or the ability to engage in conversation (Amering et al., 2012). Recognising the imperative to prevent such conditions, they sought to create a forum where persons with lived experience, relatives, and mental health professionals could convene to exchange ideas and perspectives. These seminars provided a neutral platform for dialogue, free from formal obligations (Bock & Priebe, 2005). Participants from all groups alike discovered that engaging in meaningful conversations offered valuable insights into mental health experiences.

Following the psychosis seminars, Austria emerged as a pioneering force in the trialogue model. In 1994, Vienna hosted its inaugural trialogue (Amering et al., 2002, 2012) which proved successful in fostering innovation and creating new avenues of communication. Subsequently, numerous organisations embraced this method, recognising its value in interventions. One notable initiative was the establishment of ‘The Irish Network of Mental Health Trialogues’ in 2010, dedicated to empowering communities through open, participatory dialogue (Dunne, MacGabhann, et al., 2018a, 2018b; Macgabhann et al., 2012). Concurrently, the International Network toward Alternatives and Recovery convened a pivotal trialogue conference in Toronto in 2011. This event brought together persons with lived experience, their relatives, advocates, and mental health professionals to promote innovative clinical and social practices for recovery (Amering et al., 2012). The deliberations of the conference resonated globally, leading to the establishment of over 150 trialogues across Austria, Germany, France, Poland, Switzerland, Argentina, China, and beyond (MacGabhann et al., 2018). Trialogues are also employed for training peer support workers, police officers (Wittmann et al., 2023), and in school-based projects to combat stigmatisation (Amering, 2010). Defined as dynamic discussions marked by openness and mutual appreciation of diverse experiences and perspectives, trialogues continue to evolve as vital tools in mental health dialogue and community engagement.

Trialogue meetings in the mental health field distinguish themselves from therapeutic groups by emphasising the sharing of experiences and knowledge, where participants openly discuss the challenges of care and recovery (Bock & Priebe, 2005). These meetings do not replace traditional services but serve as complementary platforms for engagement and learning. Typically convened once or twice a month, trialogue sessions are structured to include representation from persons with lived experience, relatives, and mental health professionals though they are open to anyone with an interest in mental health (MacGabhann et al., 2018). A trained moderator facilitates the discussions, ensuring that everyone has an opportunity to speak, and that the conversation remains respectful and productive. The meetings follow the guidelines of Open Dialogue, which include principles such as social network perspective and dialogism (Seikkula et al., 1995, 2006). These principles guide the facilitation process, aiming to create a supportive environment where participants can share their experiences and perspectives openly.

In everyday interactions, communication among professionals, persons with lived experience, and relatives can become so fraught with complexity that it leaves all parties feeling misunderstood, disillusioned, and isolated. Many family members and persons with lived experience perceive inadequate support from health services and often lack the agency to engage on equal terms with professionals and policymakers (Wallcraft et al., 2011). This sense of exclusion can leave them feeling marginalised and ill-prepared to handle crises effectively. In response, trialogue initiatives aim to foster active participation and empowerment among participants to instigate meaningful change. Moreover, participants seek to enhance their understanding and express their emotions and experiences. These encounters have enabled them to develop stronger communication skills and cultivate a sense of community, thereby bolstering their support networks (Dunne, MacGabhann, et al., 2018a, 2018b).

Trialogues disrupt professional paternalism and challenge the dominance of medical expertise by valuing lived experiences, fostering mutual understanding, and promoting awareness to shift attitudes among all participants. Professionals also benefit by critically reflecting on their own practices and roles, gaining valuable insights (Amering et al., 2002; Bock & Priebe, 2005). The integration of subjective viewpoints with professional knowledge enables participants to contextualise and appreciate differences in handling similar experiences (Amering et al., 2012). Furthermore, these meetings provide a platform to discuss feelings, challenges, and experiences within mental health services, promoting deeper insights and dialogue (MacGabhann et al., 2018).

This study aims to analyse the group narratives of an initial series of trialogues conducted in Barcelona, Catalonia, Spain. The research explores trialogues as a vehicle for advancing mental health and considers the potential for expanding spaces and meetings to facilitate discussions on issues, uncertainties, and concerns within mental health services.

## Methods

### Design

In this study, a qualitative design was adopted to examine trialogue meetings. Following methodologies used in similar research (Dunne, MacGabhann, et al., 2018a, 2018b; MacGabhann et al., 2018), all meetings were audio-recorded and transcribed verbatim. This methodological choice allowed for a comprehensive exploration of the group dynamics, interactions, and shared experiences among participants in the trialogue meetings.

### Participants

This pilot study aimed to explore the initial feasibility and outcomes of trialogue meetings in Barcelona, with the intention of expanding the number of participants in subsequent groups. The pilot study included thirteen participants from a community-oriented mental health centre. Among them were three individuals who described themselves as mental health professionals (a social worker, a social educator, and a psychologist), nine individuals with lived experience of severe psychosocial distress (some of whom also self-identified as relatives), and one individual who self-identified solely as a relative. This composition reflects the essential involvement of these three groups integral to the trialogue format. Investigators adhered to the condition of not collecting detailed demographic information about the participants. Despite this, it was ensured that each session included representatives from mental health professionals, individuals with lived experience, and relatives. Each participant voluntarily agreed to take part in the pilot and was briefed on the confidentiality of the data, providing signed consent for session recordings. Establishing an environment of open communication, all participants unanimously agreed to uphold equal participation and roles within the trialogue sessions.

### Procedure

Over a three-month period, six trialogue meetings lasting an hour and a half each were conducted at a community mental healthcare centre from a service provider group engaged in a transformative project towards the Recovery model. While a neutral venue is ideal for such discussions, the healthcare centre was chosen due to its accessibility and the familiarity it provided to participants, since they were all already connected to it. This setting ensured a safe and comfortable environment, which was essential for encouraging open dialogues. Participation in these sessions was entirely voluntary, and each participant maintained an equitable role throughout. All sessions were facilitated by a moderator from a first-person mental health research organisation. The Trialogue Meetings were recorded using a mobile device, and all sessions were subsequently transcribed for analysis.

### Data Analysis

The transcriptions from the Trialogue Meetings were analysed using qualitative analysis software (ATLAS.ti). We employed Reflexive Thematic Analysis (RTA) as outlined by Braun and Clarke (2019) to identify and analyse patterns within the qualitative data. This approach emphasises the researcher’s active role in theme development and the importance of reflexivity throughout the analysis process. The use of RTA facilitated the organisation and description of themes emerging from the discussions, offering an exploratory approach to understanding the pilot experience. According to Braun and Clarke (2006), thematic analysis involves six phases. Initially, the Trialogue Meetings were transcribed verbatim. Then, transcripts were thoroughly reviewed to familiarise ourselves with the data. Subsequently, the coding process was conducted using ATLAS.ti to systematically tag segments of data. The third stage of analysis involved aggregating initial codes into broader conceptual categories through an iterative process of refinement, ultimately yielding three principal themes. In the fourth stage, we conducted a comprehensive review to ensure thematic coherence and consistency, revising and refining the themes as necessary. This process culminated in the development of thematic maps that encapsulated the entire analytical framework, incorporating all initial codes and their original labels (see Supplementary materials for detailed representations). Each theme was then named to encapsulate its core concept. To enhance the credibility of our findings, participant quotes were selected to illustrate each theme, following principles of trustworthiness in qualitative research (Kaselionyte et al., 2016). Finally, we also analysed the development of the sessions trying to capture the evolution of the conversations by analysing the frequencies of themes and subthemes in each trialogue session (see Table 1).

**Table 1:** Themes, frequencies in each trialogue meeting, and total frequencies and proportions

Topics/Meeting	1	2	3	4	5	6	N	%
Recovery process								
Feelings	9	7	20	14	19	8	77	5.7
Social Exclusion	37	7	32	10	12	5	103	7.6
Support Network	5	0	42	3	3	2	55	4.1
Independence	8	8	17	9	7	7	56	4.1
Interaction with mental health services								
Information provision	13	16	12	47	11	9	108	8.0
Professional role	28	29	26	35	42	19	179	13.3
Perceptions	15	26	16	27	38	12	134	9.9
Medication use	4	19	0	15	10	0	48	3.6
Disability	9	3	6	4	2	0	24	1.8
Rights	6	53	12	9	10	0	90	6.7
Recovery	1	7	21	8	6	16	59	4.4
Trialogue dynamics								
Complaints	0	3	2	52	15	6	78	5.8
Listening	14	14	8	42	14	18	110	8.1
Change	26	7	17	22	23	23	118	8.7
Thoughts	1	3	5	29	32	41	111	8.2

## Results

Our analysis revealed three primary themes: Recovery process, Interaction with mental health services, and Trialogue dynamics, each comprising various subthemes. In addition, the supplementary material provides a detailed breakdown of the original codes that make up the subthemes. These codes offer a comprehensive exploration of the nuanced and multifaceted nature of the discussions during the trialogue meetings. For instance, Appendix A delves into various emotions expressed by participants, illustrating the complexity of feelings associated with the lived experience of severe psychosocial distress. For a breakdown of theme frequencies across sessions and overall, please refer to Table 1. Below, we provide detailed descriptions and examples of the subthemes.

### Recovery Process

This theme encompasses the various ways in which individuals navigate their journey towards mental health recovery. The theme includes sub-themes such as feelings, social exclusion, support network, and independence. Each sub-theme highlights different aspects of the recovery journey, from the emotional experiences of vulnerability and helplessness to the practical challenges of building a support network and achieving autonomy.

#### Feelings

Throughout the trialogues, participants frequently expressed emotions related to their experiences with mental health issues. Many participants shared feelings of vulnerability, as exemplified by one participant who stated:

It is not just the system and it is not just society, it is that a person can be in a situation for many years of great vulnerability, of great fragility, of needing support to be able to make these decisions (Participant 2, male).

Others described feelings of helplessness and uncertainty:

I think that sometimes what we ask for is security and sometimes we do not have security. When you go to the doctor, you would like them to tell you right then what you have, how to fix it, and whether it will work or not. And sometimes it’s true that it’s not like that. And it’s very difficult to endure, isn’t it? To endure the uncertainty . . . Between appointment and appointment, they will call you, but you don’t know when (Participant 1, female).

#### Social Exclusion

Participants in the trialogue discussions highlighted the significant impact of mental distress on social exclusion. They emphasised feelings of isolation and loneliness, with one participant stating: ‘You felt alone, you felt isolated, you felt that nobody understood you, you felt that nobody could help you’ (Participant and organiser, male).

Participants noted that these feelings often stemmed from societal biases against persons diagnosed with mental disorders, leading them to feel marginalised and excluded. As one participant expressed: ‘Just the way they look at us on the street is enough. That is the first marginalisation. And after that, all the others come … they … are afraid of them, they don’t know how to treat them’ (Participant 1, female).

#### Support Network

This subtheme underscores the crucial role of support networks in the context of mental health recovery. Many participants emphasised the significance of placing responsibility on others and selecting caregivers, acknowledging it as a weighty decision. One participant reflected on this complexity:

A debate was generated about how in certain life situations we delegate responsibility for certain actions... to a professional or family member because we are not capable or do not want to bear this responsibility ourselves. It’s challenging because it can lead to conflicts. After entrusting this responsibility, this trust in another person... perhaps after a week or a month, I may not agree with what this person decides or does for me (Participant 3, male).

Participants also discussed the emotional burden and practical challenges faced by relatives in supporting individuals with lived experience. One participant articulated concerns about the future care of their loved one:

This worry about what will happen to my son when I’m gone, right? And it’s an important weight... and of course you have had that child that you love very much but there may be a brother that it might be a conflict for him... because sometimes it is a sacrifice... it is a person that needs a lot of attention (Participant 8, female).

#### Independence

Participants in the trialogue discussions emphasised the theme of promoting autonomy. They underscored the importance of individuals having the ability to make their own decisions and not being overly reliant on others. One participant stated: ‘But a person, when they enter a place, needs to have a minimum level of autonomy and choice. They can’t constantly be at the mercy of decisions made by others’ (Participant 4, male).

Moreover, participants advocated for an active role in their own treatment and care, emphasising the need for individuals to play a proactive role alongside professionals and relatives:

But also, to expect the person we are serving to demonstrate a certain capacity... a more proactive role. It is not only about the professional’s role, but also the role of the family member and the service users themselves (Participant 2, male).

### Interaction with Mental Health Services

This theme focuses on the experiences and perceptions of persons with lived experience in their interactions with mental health professionals and systems. It includes sub-themes such as information provision, professional role, perceptions of the mental health system, medication use, disability, and rights. These sub-themes illustrate the complexities and challenges faced by persons with lived experience, including the need for clear information, empathetic professional conduct, and respect for their rights.

#### Information Provision

Information emerged as a significant theme during the trialogue meetings. Participants frequently highlighted a lack of information regarding diagnoses, treatments, and other aspects of mental health care: ‘They have the diagnosis since eight, nine, twelve years and very few people are able to explain what exactly they have. Some can name it, but neither can explain what it is and less with medication’ (Participant 2, male).

Many participants argued for comprehensive information sharing:

The patient should know what he is taking, that he is being given information about his illness and if he wants more information where can he go, where can he find the information, where can he study this information? The person has to know. I think that is a right. You should have right to information (Participant 4, male).

Participants linked information to decision-making capacity, suggesting that insufficient information could limit service users’ ability to advocate for themselves:

Then, if a patient does not know in a clear way what is happening.... He will not be able to change anything. Also, in an effective way. Because he does not know and does not have the information of what is really happening (Participant 4, male).

#### Professional Role

The role of professionals within mental health services emerged as the predominant category in the trialogue meetings. Participants addressed several aspects of professional conduct and challenges faced by mental health practitioners. Some professionals were described as adopting an overprotective attitude, potentially undermining service users’ independence and decision-making abilities. Others were perceived as authoritarian:

‘Sometimes you arrive and feel like you say stuff and he does not listen much; he explains a bit. He just needs to comply with a protocol, he is not sure you understand, he does not enter in a negotiation with you... He says what you have to take and do and to be quick because he has another patient waiting’ (Participant and organiser, male).

Participants noted a lack of sensitivity among some professionals, leading to dehumanised interventions:

There is a big lack of sensitivity in mental health. In an admission itself, the ones of the ambulance come at you, the police come at you, they enter into the house, well it seems that they come looking for a criminal and it is a person that is ill. It should have to be done differently... And with more tact.... Because the patient is already sick, is afraid, among all these people, what are you going to do? For me it is very traumatic. (Participant 1, female).

The discussion emphasised the importance of professionals developing sensitivity and empathy: ‘Clean admission, I do not think there is any. However, there are some better than others. Maybe because the person who has the ambulance has an amazing empathy and dominates the subject and finds a contact with the person’ (Participant 6, male).

#### Perceptions of the Mental Health System

Participants shared their opinions of the mental health system. Several key themes emerged:

Current mental health services were perceived as rigid and outdated: ‘We use the protocol as a safeguard. We see through the protocol. The protocol says something and then we do not leave from there. It’s like a very tight suit’ (Participant 4, female).

Participants criticised the system’s excessive focus on mental illness:

The problem is that people, or psychiatrists, focus too much on mental illness... but they do not pay attention to other things. A person is not just their mental illness. And in fact, a person is not their mental illness (Participant 4, male).

The lack of adequate staffing and resources was a recurring theme: ‘Yes, but of course you need personalised attention, and personalised attention does not exist. It is a utopia’ (Participant 1, female).

Despite the criticisms, some participants acknowledged positive aspects of the mental health system:

And from the conversations we have where I work, there’s a very clear divide. There are some people who have lived and live mental health services as allies... as a space where I can share what happens to me, they lend me a hand... they listen to me (Participant 3, male).

#### Medication Use

Medication use was a significant topic of discussion. Participants addressed several aspects of medication prescription and use in mental health services emphasising the need for responsible prescription and thorough follow-up:

When a person prescribes a medication, they have to see what they are doing... they cannot leave you like that, at least during the first days. They cannot leave you in a room... they have to assume the risks of what they are doing (Participant 1, male).

The importance of informing service users about their medication was highlighted: ‘If a doctor wants to give medication. The first thing they have to do is to inform and try to convince the patient that it’s good’ (Participant 4, male).

Some persons reported lying to their doctors to reduce medication doses:

‘They’ve got me all medicated, and I talk to a psychiatrist who tells me no... that I have to keep taking the same because I’m not improving, I keep hearing voices and all that... and... I want to fight with him—well, not fight, but negotiate with him to lower my medication because I feel like it’s affecting me somehow.” And someone else asked, “Is it the same for you?” And he said, “Now I take much less.” “And what did you do?” “I just said yes to everything.”’ (Participant 4, male).

#### Disability

Disability was the least predominant topic in the trialogue. In these narratives participants explored various aspects of this complex issue. According to professionals, the primary purpose of legal measures, including incapacitation, which was still a valid legal procedure in Spain at the time of this study, is to protect individuals and minimise risks:

If not, maybe it’s a person who has money and each day they withdraw money from the bank and wastes it. Well, then the parents or the person who tutors them, will ask for incapacitation so they do not do it (Participant 1, male).

However, participants noted that incapacitation can easily lead to paternalism and overprotection: ‘But aren’t you making him never able to learn? I mean, you protect him so much that maybe in some way it is impossible that this person learns because you had him infantilised all his life (Participant and organiser, male).

#### Rights

The rights of individuals with lived experience of severe psychosocial distress emerged as a significant issue in the trialogue meetings. Participants explored various aspects of rights violations and protections in mental health care.

Participants noted that experiencing or perpetrating rights violations is a common occurrence:

It is one thing that the people who are here with our different roles and our different experiences have happened to us... and it will probably happen to us, I mean, we have exercised it, and someone probably will do it to us. (Participant and organiser, male).

Some participants argued that rights violations can sometimes be justified for protection: ‘Rights are violated, but to take care of other people… and this is like ‘I do not respect your rights, but I do it for your own good’. (Participant and organiser, male).

#### Recovery

Recovery was a significant topic in the trialogues. Participants emphasised several key aspects of the recovery process. They stressed the importance of ongoing support and rehabilitation: ‘“I transitioned from child to adult services… moving from adult services to… creating a resource so that, when a person breaks or shatters into a thousand pieces, they can somehow be supported to… put themselves back together again’ (Participant 6, male).

The importance of proper continuity in care was highlighted:

Well in that recovery time you have maybe the doctor who makes you go maybe at the beginning every week and then every fifteen days and then once a month and then once every two months... Because if there is not that follow-up the person can really return to relapse and then you find yourself saying ...mmm and now what? (Participant 1, female).

### Trialogue Dynamics

This theme captures the unique aspects of the trialogue meetings and their impact on participants. It includes sub-themes such as complaints, listening, change, and thoughts. These sub-themes reflect the dynamics of the trialogue process, including the expression of grievances, the importance of active listening, the discussions about change, and the overall atmosphere of the meetings.

#### Complaints

Many participants indicated that trialogues helped them formulate complaints. They emphasised the importance of identifying what is failing: ‘You had not filed a formal complaint as proposed, then if you have a space where, with professionals, you could discuss the issues you encounter (…) Well, in fact, that’s what we’re doing.’ (Participant 1, female).

They felt that the current grievance system was unhelpful, and some complaints did not yield results:

In this system, if you complain, they tell you, you are a sick person ... And if not, they put it in a mailbox. A mailbox is not a person that cannot reassure you. A mailbox is an object. I have the feeling that you are putting the paper in an object, not in a person. (Participant 4, male)

#### Listening

Listening was an important aspect of the trialogue. Participants often felt a lack of listening from mental health services: ‘We have taken for granted not to listen. We have taken the habit of labelling; we have taken the habit of medicating (…) but we have lost the ability to listen.’ (Participant and organiser, male).

In contrast, participants mentioned that trialogues allowed them to foster listening and understand different points of view: ‘Sometimes the needs of people do not adapt well to the service that is given. Then it is good to know what people think, what others think, the realities of some, and the realities of others.’ (Participant 1, female).

#### Change

Change emerged as a key theme during the trialogue discussions. Participants emphasised that the experience sparked conversations about change and the challenges it posed, particularly regarding the availability of resources and the breadth of its impact:

I think it’s a cultural change and that it’s not a matter of time either (...) they are the ones that are really hard because you can hire a professional advisor, you can raise the ratio, you can do whatever but ... This is relatively simple, it takes money, but ... cultural changes of saying: ‘I have to put myself in another way in front of the patient,’ this, or ‘I have to leave the clinic and go see their environment’ or ‘I have to go to your home,’ this type of change is very difficult, I believe. (Participant 6, male)

Change is everyone’s responsibility:

‘But we are actors in this system. Users, relatives, professionals, each with their part of responsibility, and we have to consider how we do it.’ (Participant 3, male).

#### Thoughts

Participants reflected on the trialogic experience discussing the atmosphere surrounding it. Members felt confident and free in equal conditions: ‘Because it’s true that the group experience of coming here as equals, even though we are all in different places… is enriching, I think. And emotionally, as you said… it’s positive for everyone.’ (Participant 6, male).

They felt it was easy to communicate and listen to each other on the same level:

It is not common for us to have this space and to be able to communicate this way... we are not used to it. We are used to talking with professionals or with users and patients. However, I have transferred that to a level where we all start from the same point: being people. In some way, we can talk about everything that has been spoken. It is a bit easy because I have been able to be spontaneous and myself. Not the professional but myself. That is why I thought it was easy for me and we are very comfortable. It is also a reset for us. (Participant 2, female)

### Trialogue Development

The meetings were perceived as comfortable spaces fostering open communication. Participants generally expressed ease in setting aside differences and viewing themselves as equals. Active listening and empathy characterised the interactions. The trialogue meetings provided an opportunity to understand and appreciate diverse perspectives related to the mental health system. Participants appreciated the flexibility of the trialogue, which afforded them freedom to participate and transcend institutional norms. Despite occasional disagreements, respect remained a fundamental characteristic of these trialogues. Participants maintained an open attitude toward one another:

‘You defend yourself. Yes, it’s human. If they attack me, I defend myself, right? Or if I “feel attacked”, in quotes, it was not an attack, or anything.

- Well, the questioning.

- Yes, exactly.

- And those two things happened at the end, and lengthened the session a bit.’ (Participant 4, female; Participant 2 male; and organiser, male).

As it can be observed in Table 1, over the course of the six meetings, these themes evolved significantly. Initially, participants primarily expressed feelings of vulnerability and social exclusion. However, as the trialogues progressed, engagement deepened, and the atmosphere became increasingly dynamic. The focus gradually shifted towards exploring support networks and emphasising the importance of independence. By the later sessions, discussions had evolved to address more complex themes, such as professional roles within mental health services and systemic challenges. This progression reflects participants’ growing confidence and deeper involvement, highlighting the transformative potential of the trialogue process. The dynamic interplay between topics, such as the professional role and perceptions of the mental health system, highlighted the interconnectedness of the themes and the transformative potential of the trialogue methodology.

## Discussion

This study explored the first pilot experience of trialogues in Catalonia, an initiative rooted in the Recovery model. The collaboration between persons with lived experience, relatives and mental health professionals fostered a new vision of the mental health system, aligning with the goals outlined in previous research (Amering, 2010; Amering et al., 2002; Dunne, Macgabhann, et al., 2018a, 2018b; MacGabhann et al., 2018). The findings support the potential of trialogues to challenge institutional power structures, modify attitudes and roles, and promote open communication and mutual understanding.

The three main themes identified in the results—Recovery process, Interaction with mental health services, and Trialogue dynamics—directly address the key objectives of trialogues (Mcgowan, 2012). These themes demonstrate how trialogues can serve as a platform for discussing challenges within mental health services while promoting participatory decision-making and fostering a sense of community. The progression of themes over the six trialogue meetings highlights the transformative impact of sustained dialogical practice. Initially, participants shared personal experiences and feelings of exclusion, but as trust and familiarity grew, the discussions became more nuanced and focused on systemic issues and collaborative problem-solving. This transformation illustrates the potential of trialogues to not only provide a platform for sharing experiences but also to foster critical reflection and collective action. The evolving nature of the discussions underscores the importance of continuity and commitment in dialogical practices to achieve meaningful and lasting change. The relationships between the topics discussed in the trialogue meetings underscore the holistic and interconnected nature of the dialogical process. For example, discussions on professional roles often intersected with perceptions of the mental health system, highlighting systemic issues and the need for change. Similarly, conversations about support networks and independence were closely linked to themes of Recovery and Rights. The trialogue methodology facilitated these connections, enabling participants to see the broader context of their experiences and the systemic factors at play. This approach not only enriched the discussions but also empowered participants to envision and advocate for meaningful changes in the mental health system.

The encounters provided ideal conditions for trialogues to occur. Most participants had an active role and contributed to theme proposition. Although some individuals spoke more than others, members generally felt they were on equal footing. The meetings promoted equalitarian relationships, with mutual respect among all participants (Mcgowan, 2012). These groups strengthened relationships between all three parties involved in the mental healthcare context. Participants reported feeling supported and actively listened to. They were able to discuss their feelings and experiences, which decreased misunderstandings and promoted better communication. The encounters were perceived as providing a confidential atmosphere and empathic care (Macgabhann et al., 2012). Moreover, members benefited from discussing distressing experiences during the trialogues (Amering et al., 2002). Professionals indicated that the meetings helped them gain a broader insight into their practice within the mental health system. Group members felt comfortable with the participatory approach. Furthermore, the freedom to participate facilitated different forms of relationships compared to those typically found in clinical settings, which are shaped by distinct roles, expectations, power imbalances, and pressures. Although members discussed problems and complaints about the mental health system, trialogue meetings sought to go beyond this. The aim was not to assign blame but to seek solutions. The exercise questioned different perspectives of the mental health system and proposed alternatives for change (Wallcraft et al., 2011).

However, some limitations should be acknowledged. Some participants reported not fully understanding the purpose of the trialogues, highlighting the need for clearer objectives in future sessions. Others expressed that more structured goals would have been helpful. For future trialogues, it will be important to clarify participants’ expectations from the outset (Kaselionyte et al., 2016). The relatively low and inconsistent participation, while allowing for flexibility, could create barriers to delving deeper into certain issues. These challenges should be addressed in future trialogues to enhance participant engagement. Another limitation of this study is the absence of detailed demographic data on participants, which was a condition set by the organising service provider group to protect participant anonymity. While this restricts the ability to accurately describe the sample characteristics, we ensured that each session included individuals with lived experience, relatives, and professionals. Future studies should strive to balance participant confidentiality with the need for comprehensive demographic reporting.

Despite limitations, this pilot experience supports the broader implementation of trialogues. By providing a platform for equal participation and open communication, trialogues can contribute to breaking down stigma, enhancing mutual understanding, and promoting more inclusive mental health services. The initiative proved both worthwhile and sustainable, demonstrating the potential for trialogues to become an integral component of community-based mental health initiatives. Mental health organisations should consider incorporating trialogue meetings into their regular practice to promote a more inclusive and participatory approach to care. Training programs for mental health professionals should also emphasise the value of lived experience. Policymakers should support initiatives that facilitate community-based mental health initiatives, recognising the potential of trialogue meetings to transform mental health services and reduce stigma.

Future research should focus on the long-term impacts of trialogues on participants and the broader mental health system. Exploring ways to increase consistent participation and clarify objectives could enhance the effectiveness of future trialogues. As this approach continues to evolve, it has the potential to play a significant role in transforming mental health discourse and support systems in Catalonia and beyond. Additionally, exploring the specific mechanisms through which trialogue meetings facilitate change, could further our understanding of best practices for implementing them across diverse settings.

## Conclusion

In conclusion, this pilot experience represents a promising step towards a more inclusive, participatory approach to mental health care. While challenges remain, this study provides a foundation for future trialogue implementations and research in this innovative field, contributing to the broader goals of recovery-oriented and community-based mental health care.

## Supplementary Information

Below is the link to the electronic supplementary material.

Supplementary file1 (PDF 1721 KB)
