# Correction: A Qualitative Analysis of Trialogues Between People with Lived Experience, Their Relatives, and Mental Health Professionals

**DOI:** 10.1007/s10597-024-01436-7

**Published:** 2024-12-30

**Authors:** Francisco José Eiroa‑Orosa, María Incera‑Rosas

**Affiliations:** 1 Section of Personality, Evaluation, and Psychological Treatment, Department of Clinical Psychology and Psychobiology, School of Psychology, Institute of Neurosciences, University of Barcelona, Barcelona, Catalonia Spain; 2 First Person Mental Health Research Group, Veus, Catalan Federation of First Person Mental Health Organisations, Barcelona, Spain

**Correction to: Community Mental Health Journal**

10.1007/s10597-024-01402-3

In the original version of this article, unfortunately contained an error in supplementary material.

In supplementary material, the Appendix N was repeated erroneously as Appendix O. Now, the supplementary file has been replaced with corrected Appendix O.

The original article has been corrected.

## Electronic Supplementary Material

Below is the link to the electronic supplementary material.

Supplementary Material 1

