# Supplementary material for: A Qualitative Analysis of Trialogues Between People with Lived Experience, Their Relatives, and Mental Health Professionals
Source: Community Ment Health J. 2024 Dec 6;61(4):764–74. doi: 10.1007/s10597-024-01402-3 (PMC11968523; doi:10.1007/s10597-024-01402-3)

Appendix A. Feelings

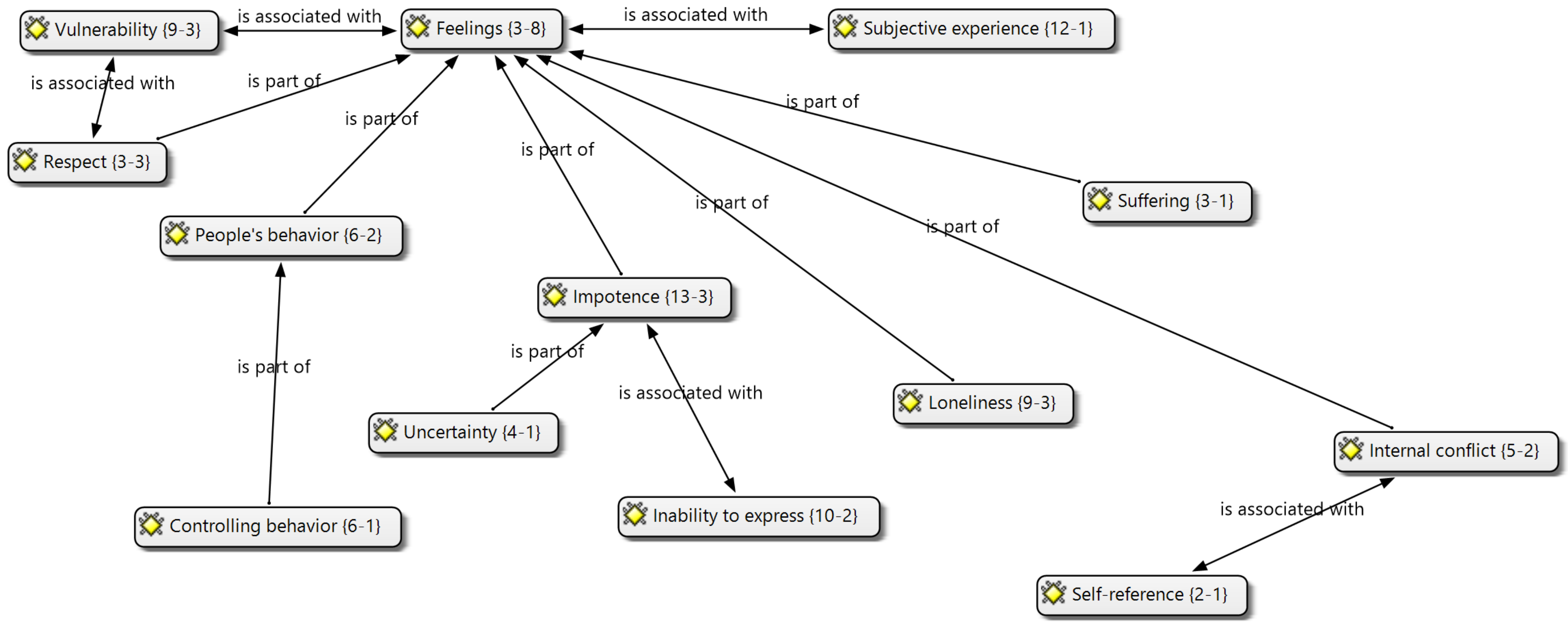

Appendix B. Social exclusion

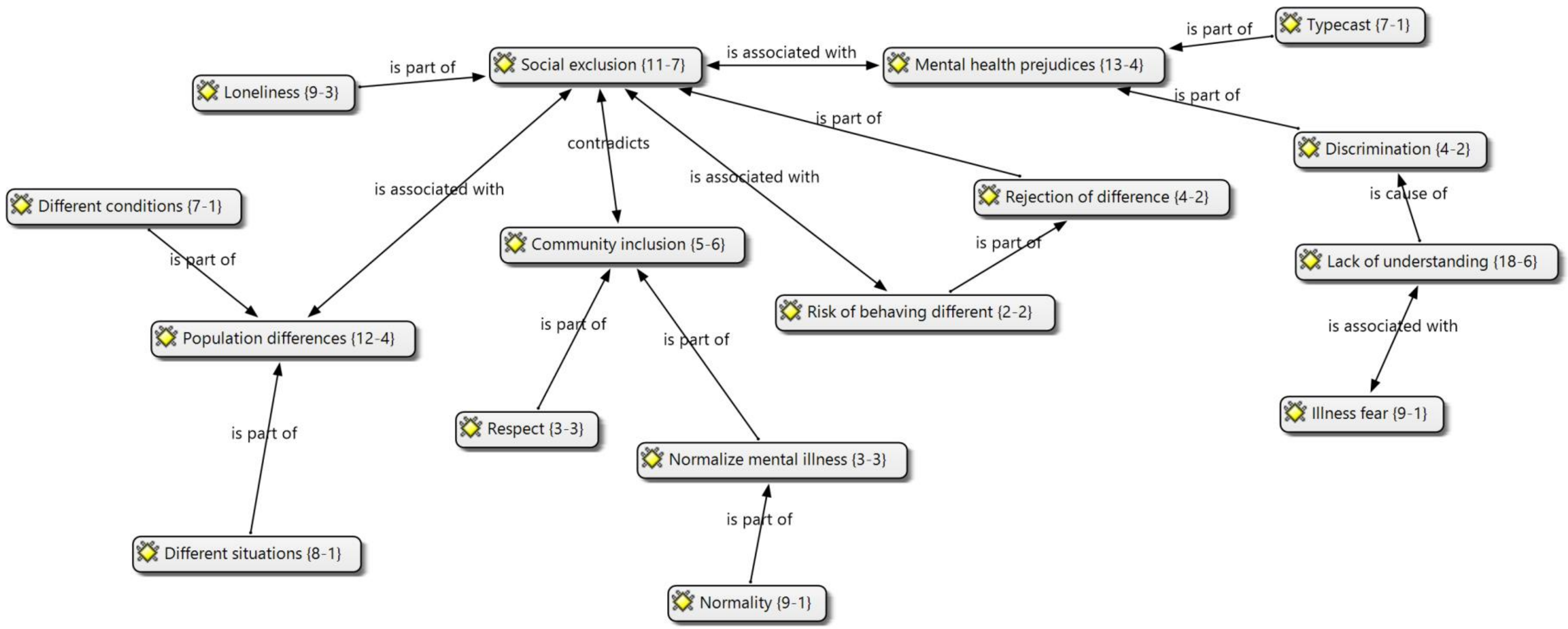

Appendix C. Support Network

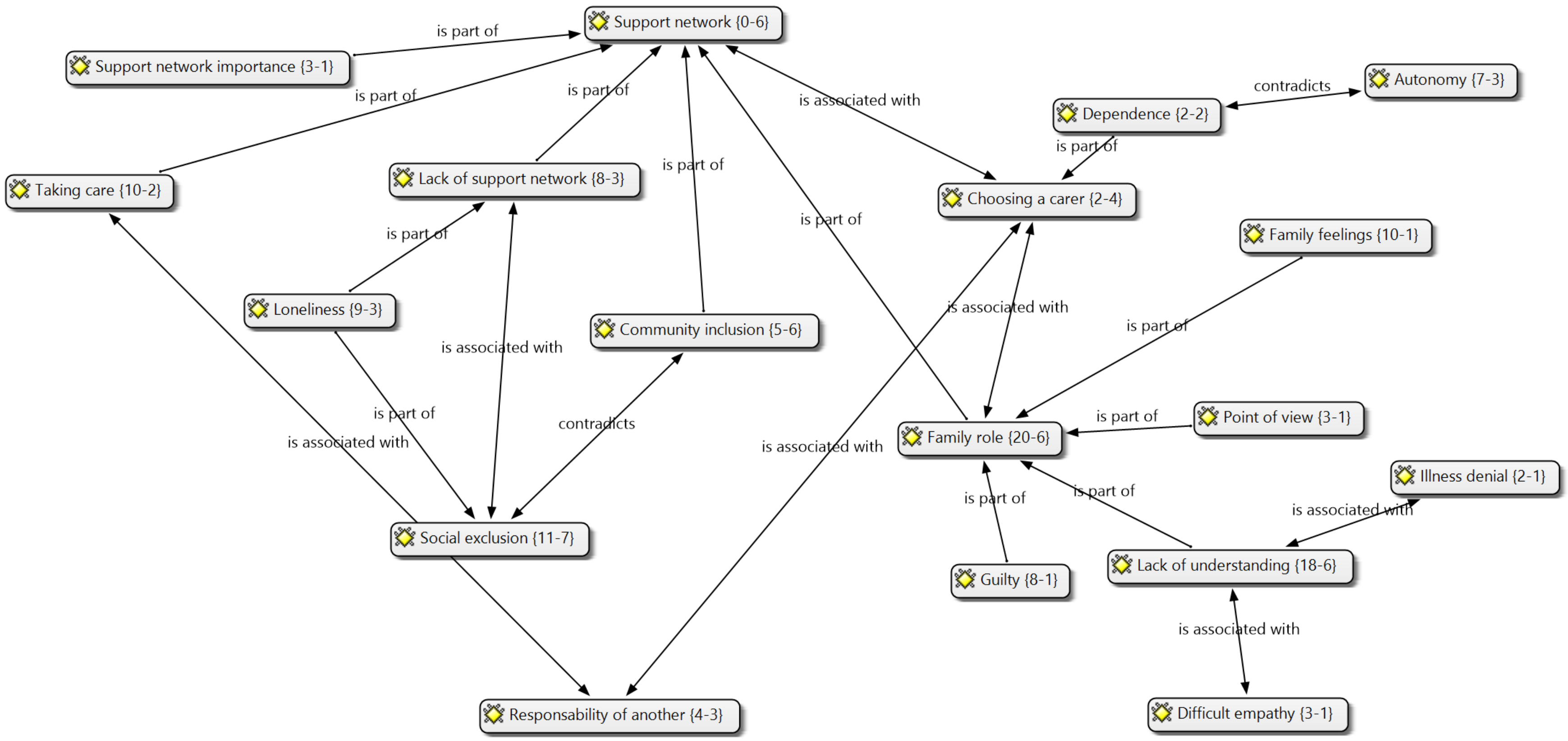

Appendix D. Independence

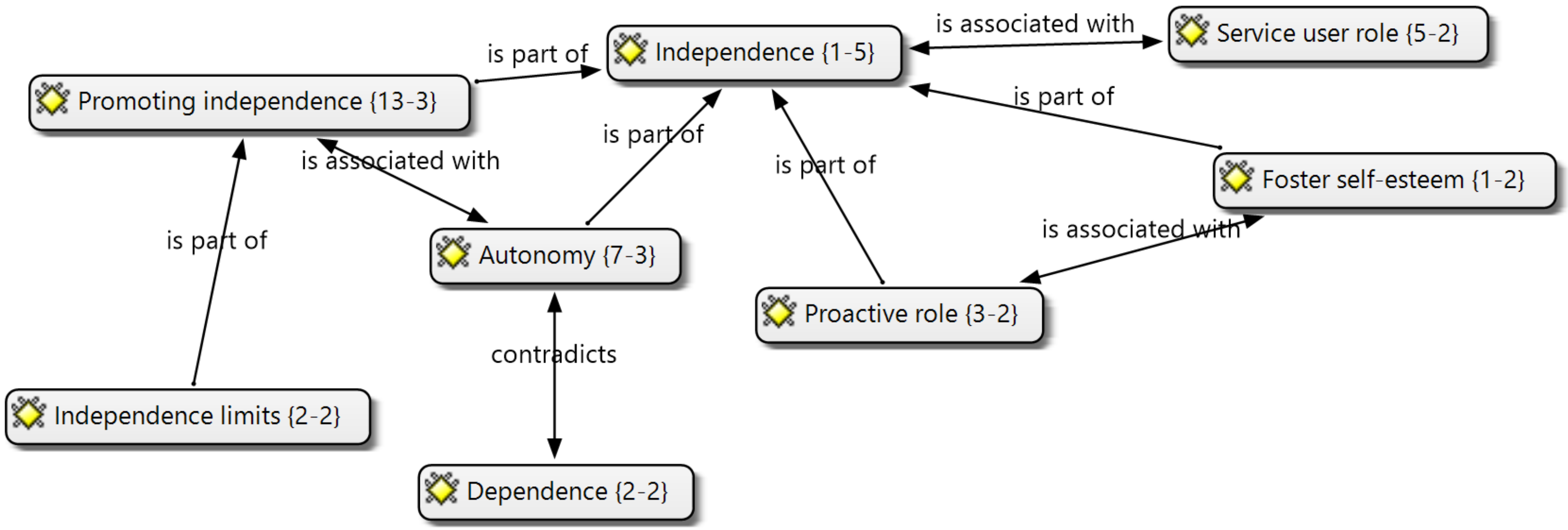

Appendix E. Information provision

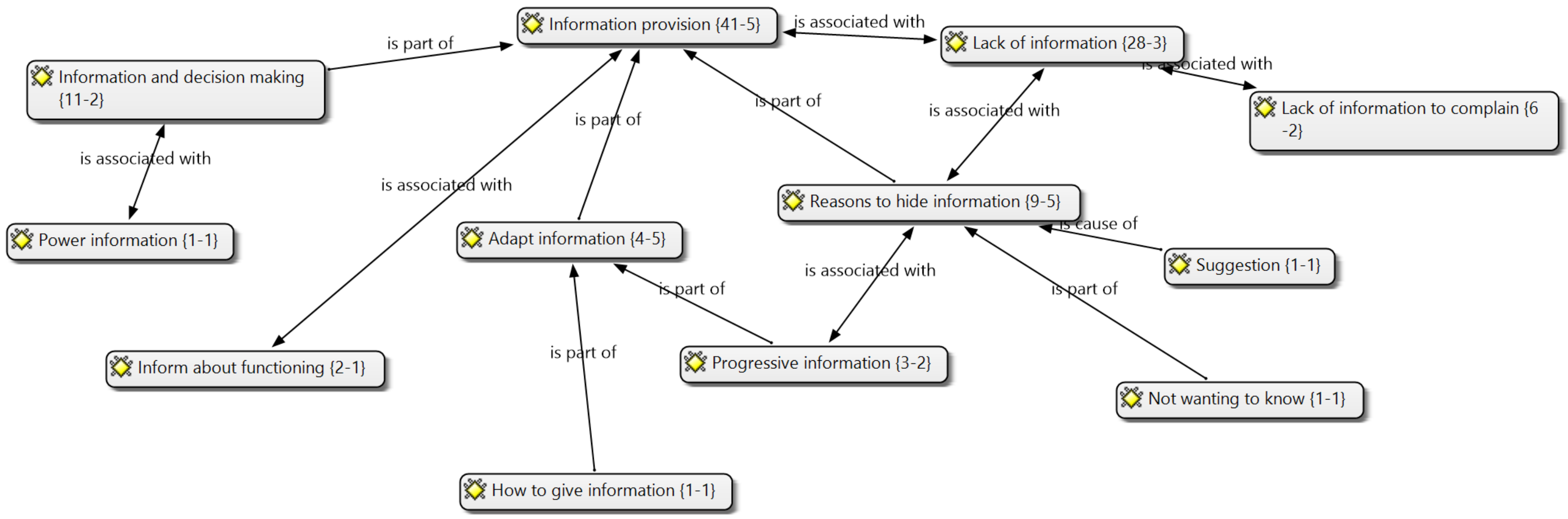

Appendix F. Professional role

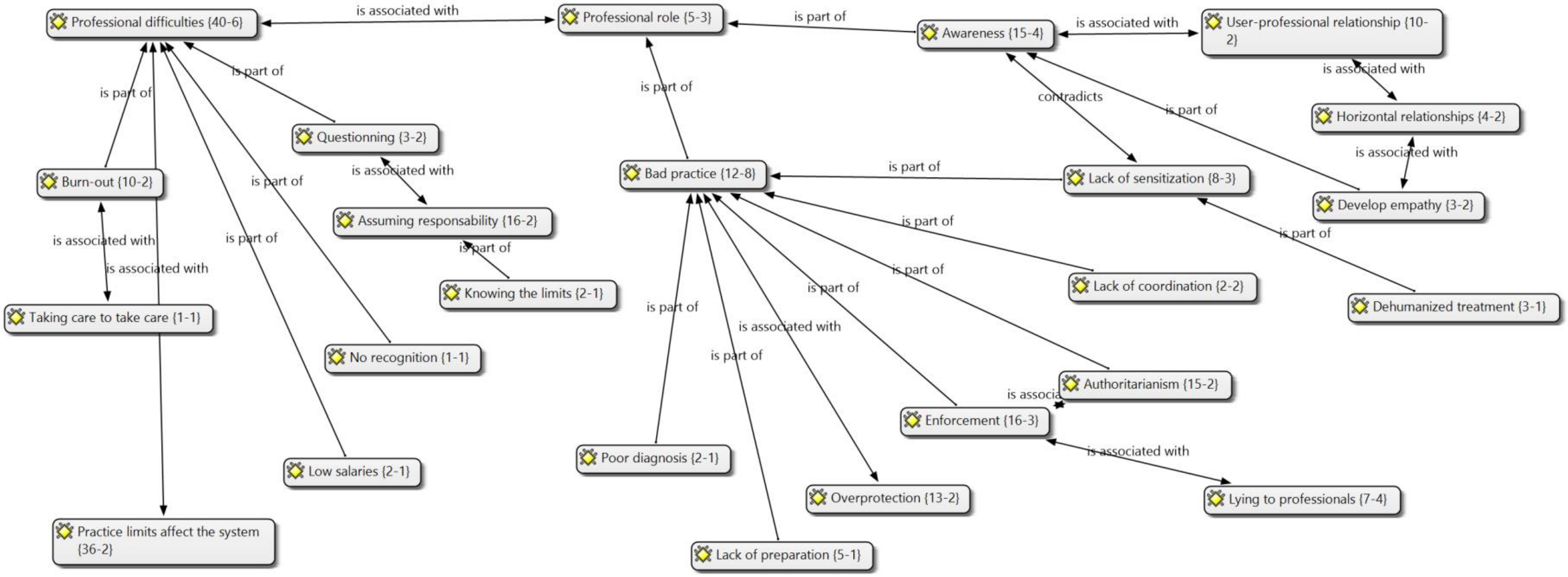

Appendix G. Perceptions of the mental health system

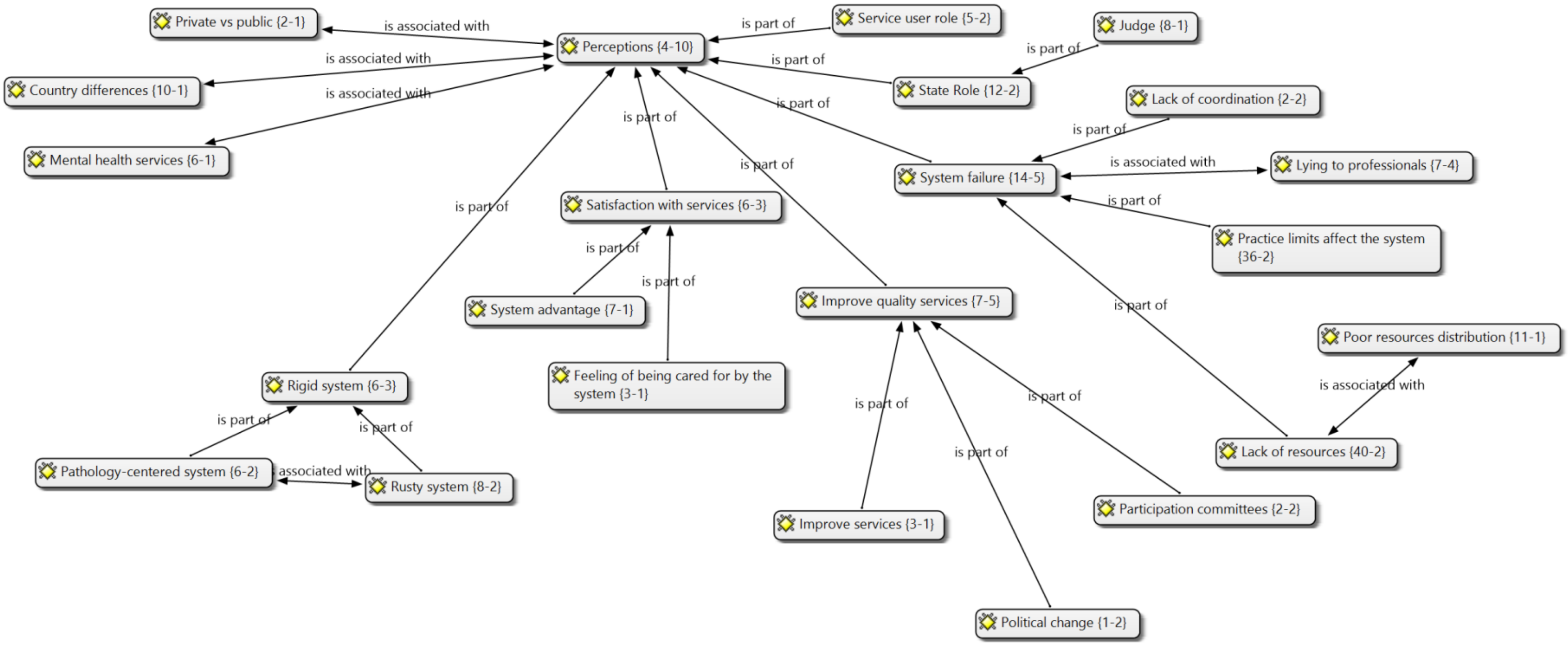

Appendix H. Medication use

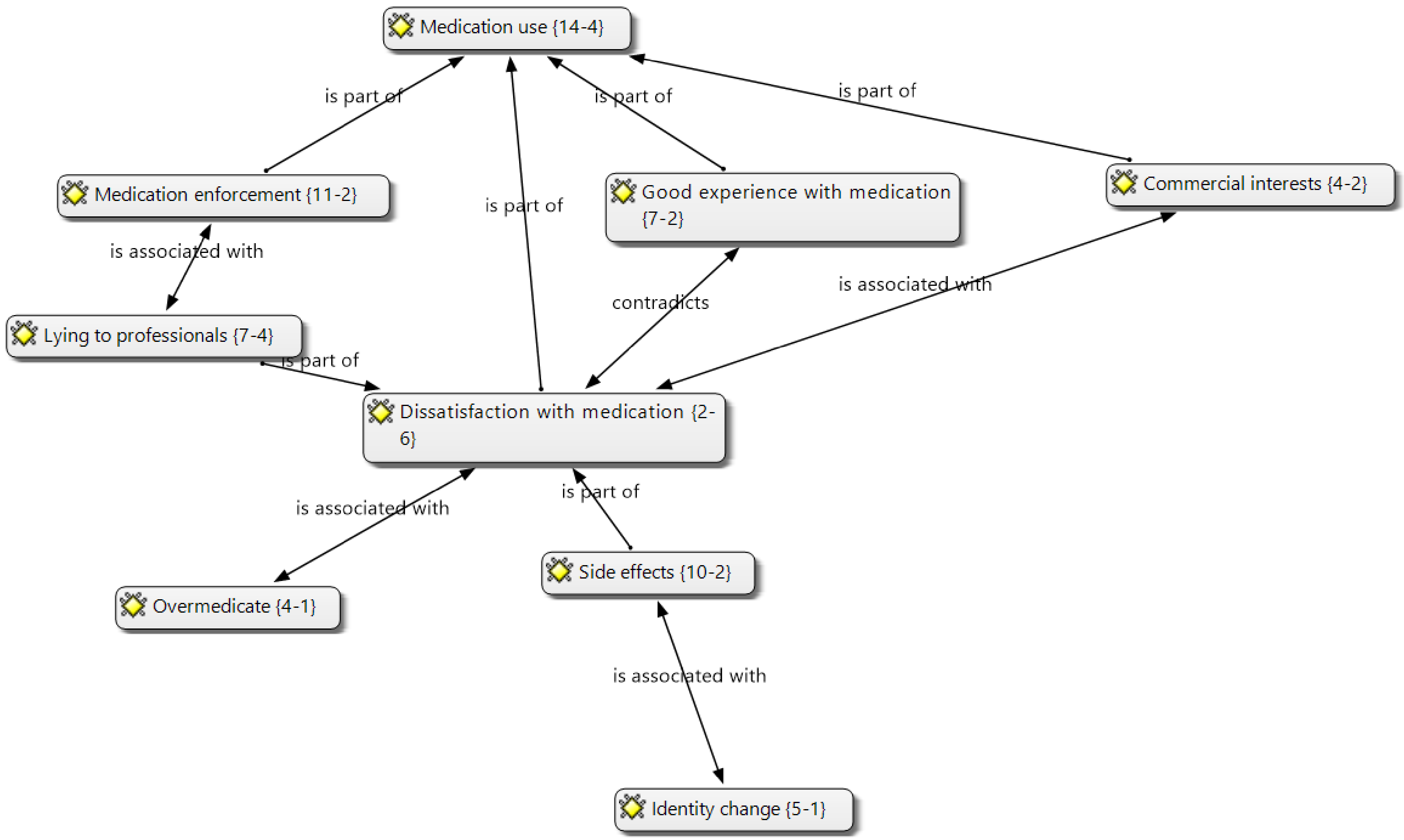

Appendix I. Disability

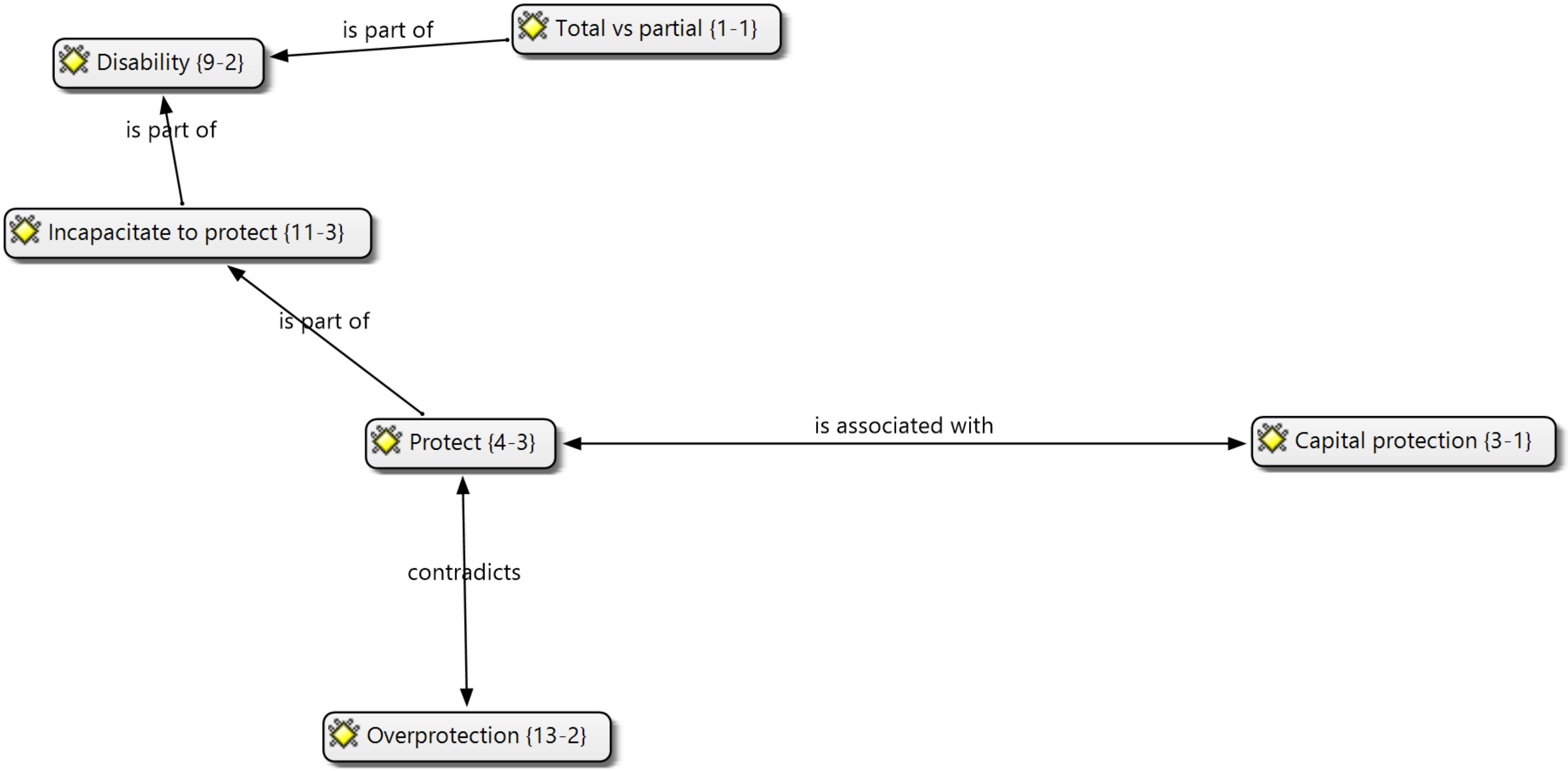

Appendix J. Rights

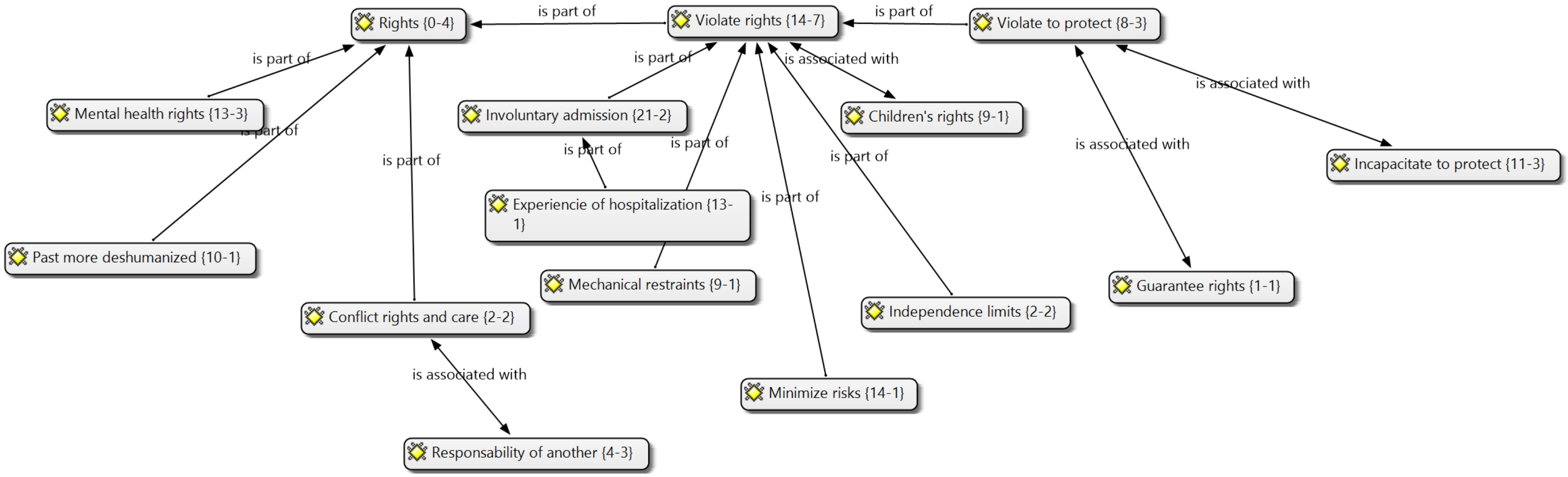

Appendix K. Recovery

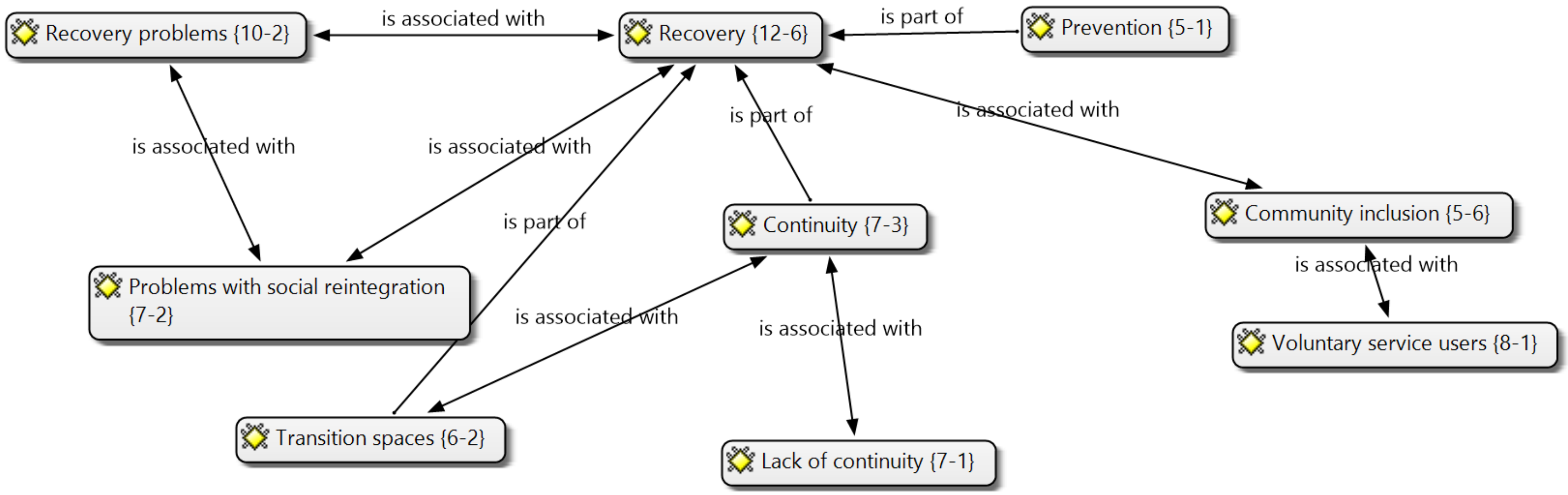

Appendix L. Complaints

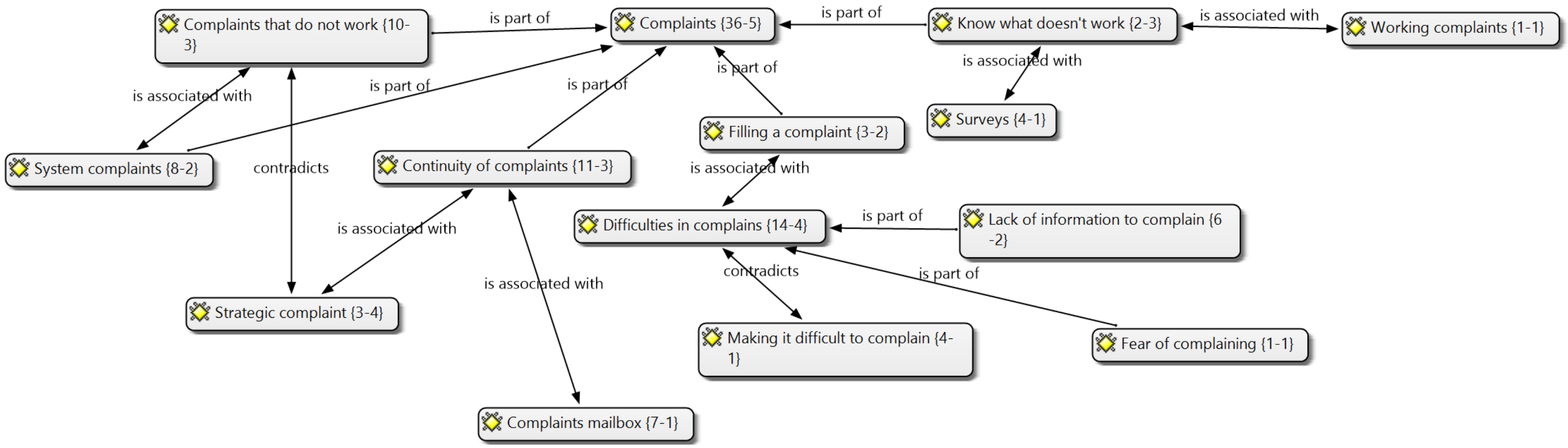

Appendix M. Listening

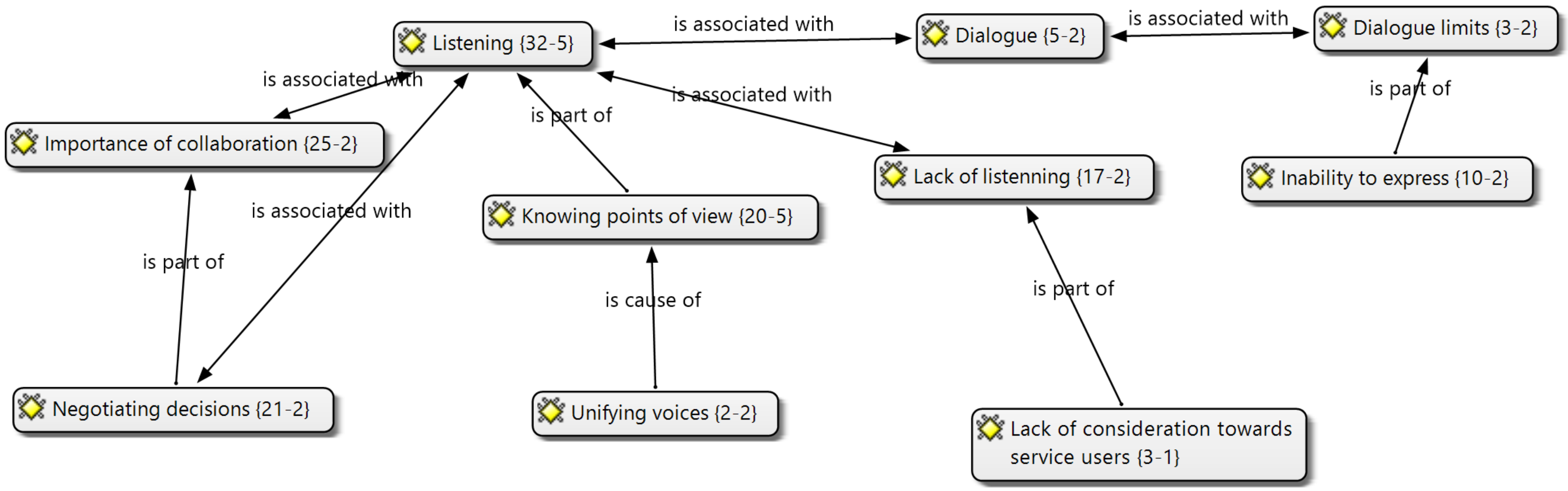

Appendix N. Change

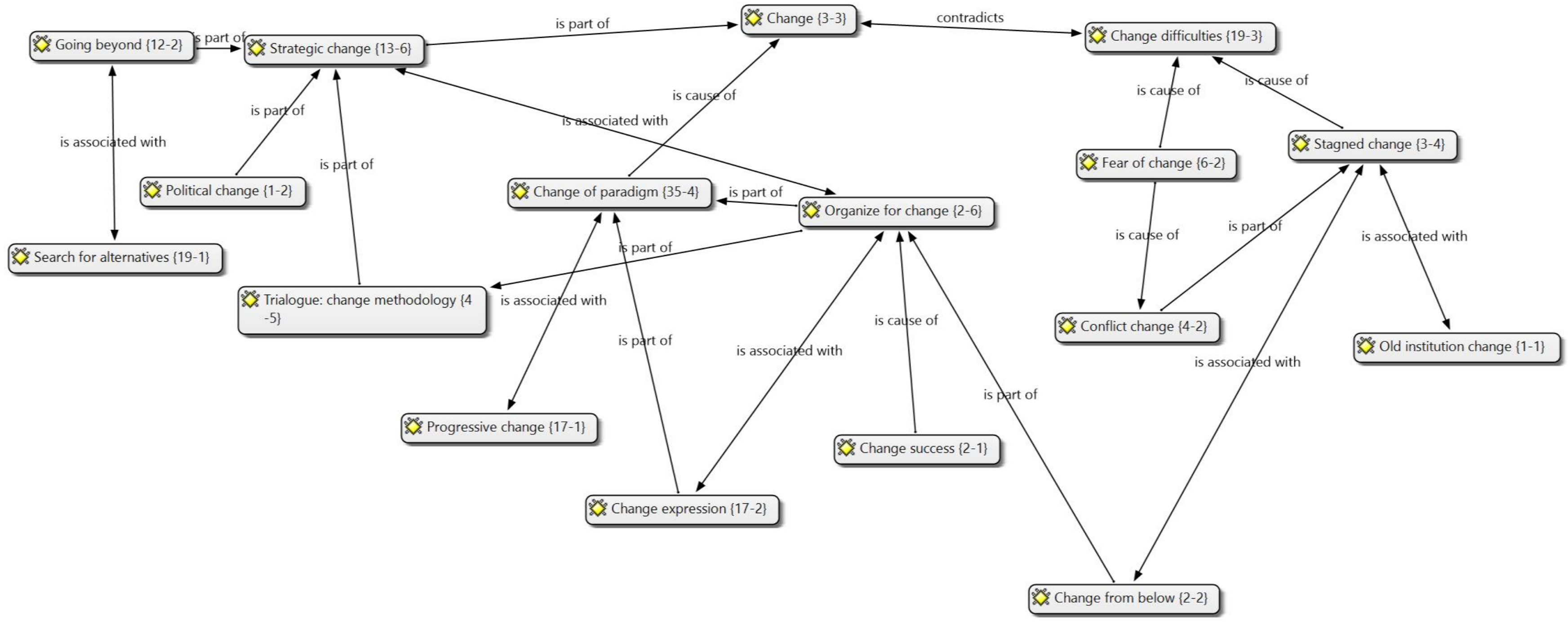

Appendix O. Thoughts

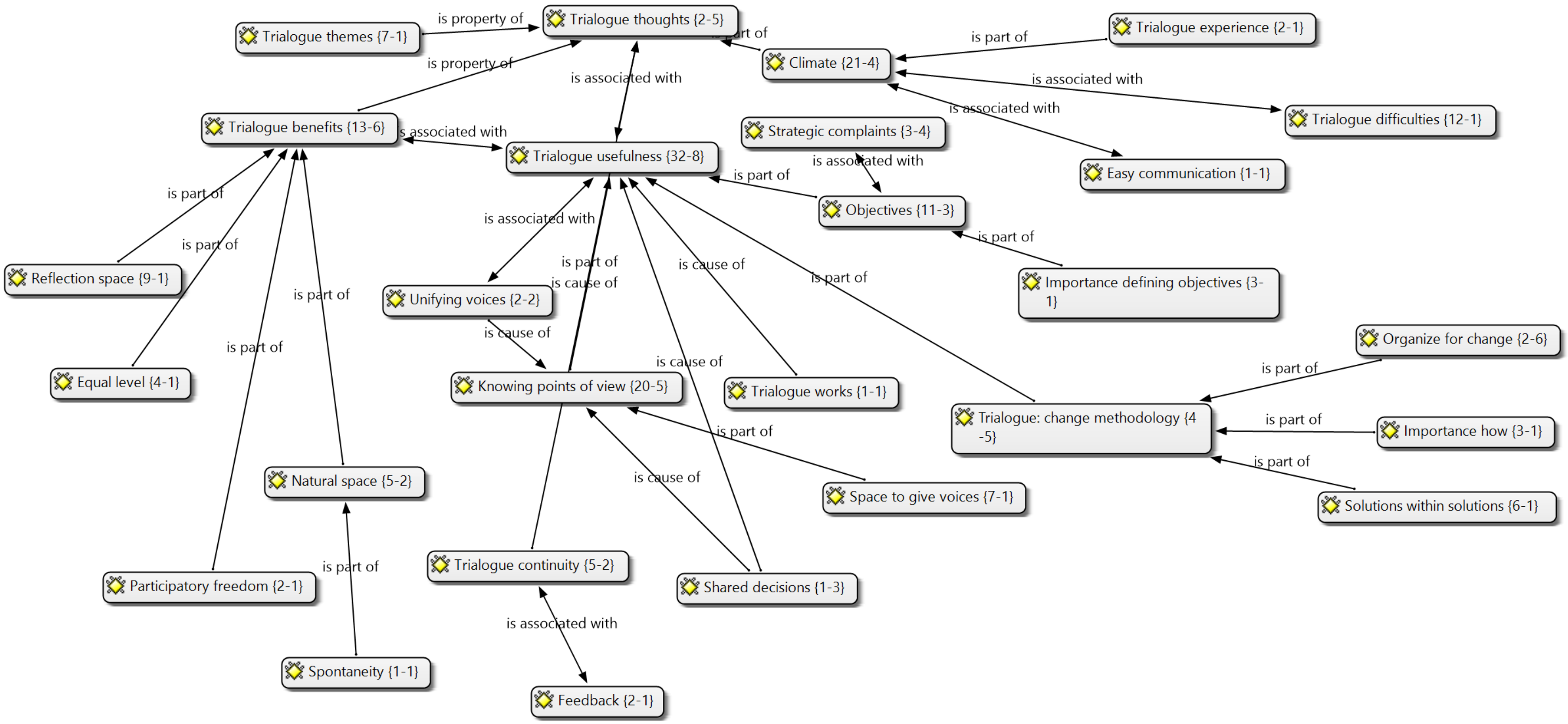

Supplement: Supplementary file 1 — Supplementary file1 (PDF 1721 KB) [file 10597_2024_1402_MOESM1_ESM.pdf]
